# Patient-specific modeling of stroma-mediated chemoresistance of pancreatic cancer using a three-dimensional organoid-fibroblast co-culture system

**DOI:** 10.1186/s13046-022-02519-7

**Published:** 2022-10-22

**Authors:** Sebastian Schuth, Solange Le Blanc, Teresa G. Krieger, Julia Jabs, Miriam Schenk, Nathalia A. Giese, Markus W. Büchler, Roland Eils, Christian Conrad, Oliver Strobel

**Affiliations:** 1 European Pancreas Center, Department of General Surgery, University Hospital Heidelberg, Heidelberg, Germany; 2 Division of Molecular Oncology of Gastrointestinal Tumors, German Cancer Research Center (DKFZ), Heidelberg, Germany; 3 NCT partner site Heidelberg, a clinical-translational cancer research partnership between University Hospital Heidelberg and DKFZ, Heidelberg, Germany; 4 Division of Visceral Surgery, Department of General Surgery, Medical University of Vienna, Vienna, Austria; 5 Berlin Institute of Health at Charité – Universitätsmedizin Berlin, Digital Health Center, Berlin, Germany; 6 Division of Theoretical Bioinformatics, German Cancer Research Center (DKFZ), Heidelberg, Germany; 7 Present Address: Merck Healthcare KGaA, Global Research, Darmstadt, Germany

**Keywords:** Pancreatic cancer, Patient-derived organoids, Cancer-associated fibroblasts, Drug screening, Personalized oncology

## Abstract

**Background:**

Cancer-associated fibroblasts (CAFs) are considered to play a fundamental role in pancreatic ductal adenocarcinoma (PDAC) progression and chemoresistance. Patient-derived organoids have demonstrated great potential as tumor avatars for drug response prediction in PDAC, yet they disregard the influence of stromal components on chemosensitivity.

**Methods:**

We established direct three-dimensional (3D) co-cultures of primary PDAC organoids and patient-matched CAFs to investigate the effect of the fibroblastic compartment on sensitivity to gemcitabine, 5-fluorouracil and paclitaxel treatments using an image-based drug assay. Single-cell RNA sequencing was performed for three organoid/CAF pairs in mono- and co-culture to uncover transcriptional changes induced by tumor-stroma interaction.

**Results:**

Upon co-culture with CAFs, we observed increased proliferation and reduced chemotherapy-induced cell death of PDAC organoids. Single-cell RNA sequencing data evidenced induction of a pro-inflammatory phenotype in CAFs in co-cultures. Organoids showed increased expression of genes associated with epithelial-to-mesenchymal transition (EMT) in co-cultures and several potential receptor-ligand interactions related to EMT were identified, supporting a key role of CAF-driven induction of EMT in PDAC chemoresistance.

**Conclusions:**

Our results demonstrate the potential of personalized PDAC co-cultures models not only for drug response profiling but also for unraveling the molecular mechanisms involved in the chemoresistance-supporting role of the tumor stroma.

**Supplementary Information:**

The online version contains supplementary material available at 10.1186/s13046-022-02519-7.

## Background

Pancreatic ductal adenocarcinoma (PDAC) has become the third and fourth leading cause of cancer related death in Northern America and Europe, respectively [1]. Chemoresistance represents a major challenge in the treatment of resectable and unresectable PDAC, yet systemic therapies remain largely restricted to conventional cytotoxic drugs administered as single agents or combination regimens [2]. The PDAC tumor microenvironment, consisting mostly of extracellular matrix (ECM), cancer-associated fibroblasts (CAFs), infiltrating immune cells and vasculature, plays a crucial role in driving tumor chemoresistance via diverse, not yet fully elucidated mechanisms [3]. A very prominent desmoplastic reaction driven by CAFs constitutes up to 90% of the total PDAC tumor volume [4]. This vast tumor ECM forms a physical barrier that can lead to decreased drug delivery to tumor cells [5, 6]. Several studies suggest that CAFs promote tumor progression, metastasis and therapy resistance [7]. CAF-mediated chemoprotection against gemcitabine in PDAC is thought to involve a variety of mechanisms such as induction of anti-apoptosis/pro-survival pathways in tumor cells [8, 9], alteration of tumor gemcitabine metabolism [10, 11] and release of exosomes [12].

Suboptimal tumor modeling neglecting tumor-stromal interactions is regarded as an important contributor to the high drug attrition rate of preclinically promising drugs [13]. Incorporation of stromal components into drug screening models is therefore urgently needed. PDAC tumor organoids [14] have emerged in recent years as 3D in vitro disease models able to retain the intrinsic heterogeneity and genetic alterations of the original tumors [15–17]. As PDAC organoids are amenable to clinical application and seem to reflect patient drug response [16, 18, 19], they hold great potential for personalized oncology. Yet, drug screening based on purely epithelial organoid culture models fails to consider the contribution of the patient-specific tumor microenvironment. Hence, incorporation of relevant stromal compartments to organoid cultures is a crucial step for optimization of tumor tissue modeling and drug response prediction.

Recently, heterocellular organoid cultures have aided to grasp CAF heterogeneity and dissect complex tumor-stroma interactions. For instance, two distinct yet plastic CAF subtypes, namely myofibroblastic and inflammatory CAFs, were identified using a murine organoid co-culture system [20]. Tsai and colleagues established cultures of PDAC organoids including CAFs and T cells, further demonstrating the suitability of these models for investigating the PDAC tumor microenvironment [21]. However, personalized drug screening assays using matched 3D heterotypic organoid cultures have not been reported yet.

Here, we established direct 3D co-culture models of patient-derived PDAC organoids (PDOs) and patient-matched CAFs. Using the live image-based drug assay DeathPro [22], we investigated the effect of CAFs on PDO chemosensitivity to the first-line chemotherapeutic drugs gemcitabine, 5-fluorouracil (5-FU) and paclitaxel, and elucidated transcriptional changes induced by tumor-stroma interaction at the single-cell level.

## Methods

### PDAC tumor specimens

Tumor tissue biopsies were obtained from surgically resected tumor specimens from patients who underwent primary resection at the Department of General, Visceral and Transplantation Surgery, Heidelberg University Hospital. The study was approved by the ethical committee of University of Heidelberg (ethic votes 301/2001, 159/2002, S-206/2011, S-708/2019 and S-083/2021) and was conducted in accordance with the Helsinki Declaration. All patients provided written informed consent prior to acquisition of tissue. Tumor entity was confirmed by pathological assessment of the resected specimens.

### Establishment and culture of PDAC tumor organoids

Primary tumor organoid cultures were established as described previously [16]. Briefly, tumor specimens were minced and incubated in tissue digestion medium containing Advanced DMEM/F-12 (Gibco), 200 mM GlutaMAX (Gibco), 1 M HEPES (Gibco), 1x Primocin (InvivoGen), 1 mg/ml collagenase IV (Sigma-Aldrich), 100 μg/ml DNase I (AppliChem), 1x B27 (Gibco), 1 mM N-acetylcysteine (Sigma-Aldrich) and 10 μM Y-27632 (Selleckchem). Cells were resuspended in growth factor reduced Matrigel (Corning) and were seeded as 50 μl drops in 12-well plates. After 15 minutes at 37 °C, 1 mL of organoid growth medium containing Advanced DMEM/F12, 200 mM GlutaMAX, 1 M HEPES, 1x B27 supplement, 1 mM N-acetylcysteine, 10% RSPO1-conditioned medium, 100 ng/ml FGF-10 (PeproTech), 100 ng/ml Noggin (PeproTech), 500 nM A83-01 (Tocris) and 1x Primocin was added. Organoids were passaged approximately every 7 days by dissociation using TrypLE (Gibco) for 10 min at 37 °C. In order to rule out overgrow of normal ductal cells and confirmed cancer cell origin, genomic alteration profiles consistent with PDAC were verified for the established organoid lines by whole-genome sequencing as described previously in detail (16). The success rate of tumor organoid establishment was 50%. All organoid lines were tested negative for mycoplasma contamination (Venor GeM Classic, Minerva Biolabs).

### Isolation and culture of cancer-associated fibroblasts

Primary cancer-associated fibroblasts were isolated from the tumor specimens using the outgrowth method [23]. Pieces of minced tissue generated in the process of organoid isolation were separated and incubated with fibroblast medium containing RPMI Medium 1640 (Gibco) supplemented with 10% fetal calf serum (Gibco), 200 mM GlutaMAX (Gibco) and Penicillin/Streptomycin (Gibco). Success rate for CAF culture establishment was 90%. CAFs were passaged approximately every 8-9 days and their identity was confirmed by morphological assessment and immunofluorescence staining for α-SMA. All CAF lines tested negative for mycoplasma contamination and were used for experiments within 4 to 7 passages after isolation.

### 3D co-culture of tumor organoids and CAFs

Organoids were digested into single cells/small aggregates (organoid forming units) using TrypLE Express (Gibco) supplemented with 100 μg/ml DNase I (AppliChem) and 10 μM Y-27632. Organoid forming units and fibroblasts were mixed in a 1:1 ratio. Depending on the growth rate of each organoid line, 2000-3000 organoid forming units per 10 μl of matrix was used. Cells were seeded as drops in co-culture matrix containing Matrigel and a 3 mg/ml Collagen I (Corning) gel solution mixed in a 2:1 ratio. Co-cultures were maintained in co-culture medium containing Advanced DMEM/F12, 200 mM GlutaMAX, 1 M HEPES, 1x B27, 100 ng/ml FGF-10, 50 ng/ml EGF (PeproTech) and 5% RSPO1-conditioned medium.

### Image-based drug testing and drug response analysis

Chemosensitivity of organoids in mono- and co-culture was assessed using the live image-based drug assay DeathPro [22]. Mono- and co-cultures of PDOs and PDO/CAFs were seeded into μ-Chamber Angiogenesis 96-well-plates (ibidi) as 10 μl drops in co-culture matrix and cultured with 70 μl co-culture medium. CAFs were stained with Cell Tracker Green CMFDA (Invitrogen) before seeding to distinguish them from PDOs. The drug screen included gemcitabine (Selleckchem), 5-FU (Selleckchem) and paclitaxel (Selleckchem). For drug treatment, serial dilutions (1:4 for gemcitabine and 5-FU, 1:3 for paclitaxel) were prepared by mixing stock solutions with co-culture medium. Drugs were applied 3 days after seeding and washed out after 72 hours. Each PDO line was tested twice independently. Confocal imaging was performed 0 and 120 h after drug application at similar positions. Four hours prior to each imaging cells were stained with 1 μg/ml Hoechst (Invitrogen) and 1 μg/ml propidium iodide (Sigma-Aldrich). Images were taken in a standardized way according to DeathPro image acquisition [22] using the Visual Basic for Applications macro “Autofocus Screen” [24]. Hoechst and propidium signals were acquired simultaneously, cell tracker signals sequentially to avoid spectral overlaps. Two positions were imaged per well. At each imaging position stacks of 17-18 slices with 50 μm slice distance were acquired. Overall, approximately 90.000 confocal images were acquired, from which around 5.700 maximum intensity projections were created and analyzed. Image processing and drug response analysis was performed using the DeathPro workflow as previously described [22]. Mean values of each independent replicate were used for the analysis.

### Single cell RNA-sequencing

Single cell RNA-sequencing (scRNA-seq) was performed for 3 pairs of matched PDOs/CAFs co-cultures and the corresponding PDO and CAF 3D monocultures. For each condition, 3 co-culture matrix drops of 30 μl were seeded into one well of a 12-well plate. Single cell dissociation was performed after 5 days in culture as we previously described [25]. Single-cell sequencing libraries were prepared according to the 10x Genomics Single Cell 3 v2 Reagent Kit User Guide. Libraries were sequenced in one lane per sample with the Illumina NextSeq 500 system in high-output mode (paired-end, 75 bp).

### Analysis of scRNA-seq data

Raw sequencing data were processed with CellRanger version 2.1.1 (10x Genomics), using the 10x reference human genome hg19 1.2.0 for alignment. Seurat version 3.0 [26] was used for quality control and downstream analysis. Cells with fewer than 200 genes, as well as genes represented in fewer than 3 cells, were excluded from the analysis. We also determined a maximum number of counts per gene, and a maximum fraction of mitochondrial reads, for each sample based on individual sample quality (Supplementary Fig. 2B, Supplementary Table S1). Count data was log-normalized with a scale factor of 10.000, and the 2.000 most variable genes were identified using the FindVariableFeatures function in Seurat. Normalized data were scaled with the ScaleData function.

To differentiate CAF and PDAC cells, expression data from all samples was merged and cells were assigned their identity based on established marker genes [27]; decorin (DCN) and lumican (LUM) expression was used to identify CAFs while expression of keratin 18 (KRT18) and keratin 19 (KRT19) was used to identify tumor organoid cells. For further analysis, PDAC and CAF cells, respectively, from monoculture and co-culture samples were combined using the IntegrateData function in Seurat. Dimensional reduction was performed using the umap-learn package [28]. Cells were clustered by the Louvain algorithm with a resolution of 0.2. Differentially expressed genes were identified using a Wilcoxon rank sum test. To link differentially expressed genes with biological pathways and functions, gene set enrichment analysis was performed using the MSigDB database [29, 30], excluding genes with an adjusted *p*-value > 0.05. In the CAF samples, two clusters that showed enrichment for ribosomal and mitochondrial genes, respectively, were excluded from further analyses.

To distinguish iCAF-like and myCAF-like cells, we performed principal component analysis on the expression of iCAF and myCAF marker genes [27] in CAF cells, followed by Louvain clustering with a resolution of 0.1 to obtain two clusters. Scores for different gene sets were calculated using the AddModuleScore function in Seurat, using published lists of PDAC subtype marker genes [31] and gene sets from the MSigDB database [29, 30] for EMT (HALLMARK_EPITHELIAL_MESENCHYMAL_TRANSITION) and proliferation (HALLMARK_E2F_TARGETS). Cell cycle scores were calculated with the CellCycleScoring function in Seurat. Potential ligand-receptor interactions between CAF and PDAC cells in co-culture were identified using CellPhoneDB [32]; PDAC clusters 2 and 4, which both comprised cycling cells, were combined for this analysis.

### Immunofluorescence staining

Paraffin embedded tumor tissue sections of 4 μm were co-stained with an anti-CD44 polyclonal rabbit antibody (1:150; Sigma-Aldrich, HPA005785) and an anti-HGF monoclonal mouse antibody (1:150; OriGene TA807186) overnight at 4 °C after antigen retrieval at pH 8.5. Donkey anti-mouse Alexa Fluor 555 and donkey anti-rabbit Alexa Fluor 488 conjugated antibodies were incubated at 1:200 for 1 h at room temperature. Slides were mounted using Fluoroshield with DAPI (Sigma-Aldrich). Confocal images were acquired in a Nikon confocal microscope using a 60× oil objective. Image processing was performed in Fiji (ImageJ).

## Results

### 3D co-cultures of matched patient-derived pancreatic tumor organoids and cancer-associated fibroblasts

Five patient-matched pairs of PDAC tumor organoids (PDAC-PDOs) and CAFs were established from surgically resected tumor samples at a success rate of 50%, with individual success rates of 50 and 93% for PDOs and CAFs, respectively. Expansion of PDOs of sufficient purity and biomass for downstream applications was completed approximately between 20 and 90 days, with a median of around 40 days. The clinicopathological characteristics of the patients are described in Table 1.

**Table 1:** Clinicopathological characteristics of the cases and PDO subtype

Organoid line	Sex	Age (years)	Tumor type	Presentation	Tumor location	pT	pN	cM	Grading	Subtype^**a**^
100PO	Female	70.1	PDAC	Primary	Head	T2	N2	M0	G3	Basal-like
107PO	Male	62.6	PDAC	Primary	Head	T3	N2	M0	G2	Basal-like
112PO	Male	65.1	PDAC	Primary	Body	T3	N1	M0	G3	Classical
121PO	Female	75.3	PDAC	Primary	Body	T2	N2	M0	G2	Classical
125PO	Female	80.2	PDAC	Primary	Head	T2	N2	M0	G3	Basal-like

^a^Determined from bulk RNAseq data of PDOs using PurIST [33]

**Fig. 1:**
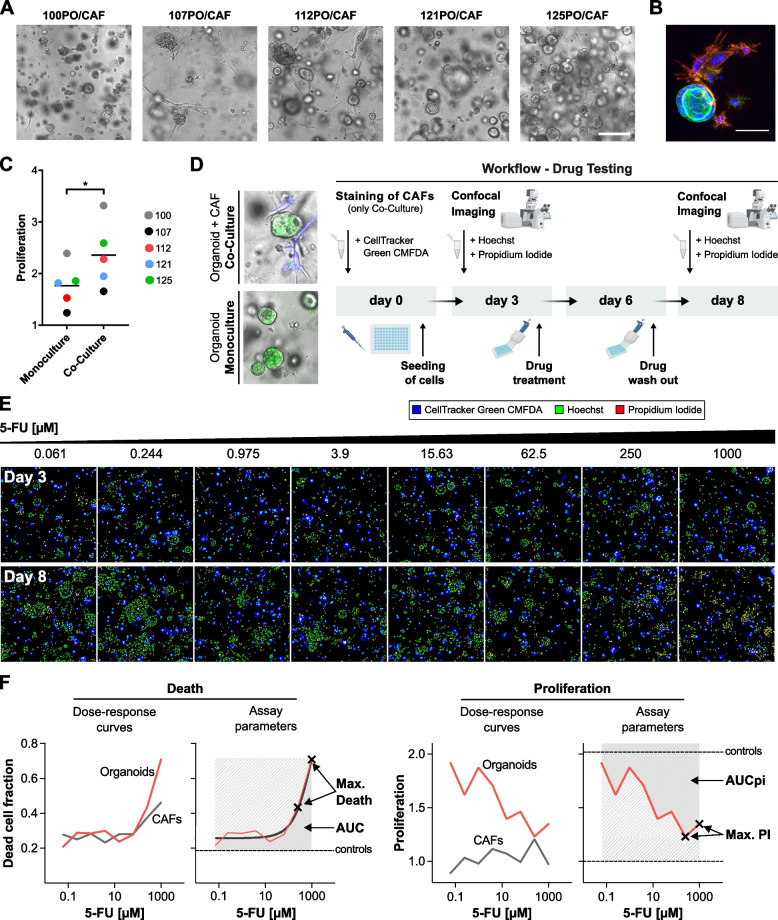
Co-culture of PDAC organoids with CAFs and image-based drug testing to de-convolve CAF and organoid responses. **A** Brightfield images corresponding to the five established direct 3D co-cultures of the patient-matched CAFs and PDAC-PDOs. Scale bar: 250 μm. **B** Co-culture of matched CAFs and PDAC-PDOs stained for the CAF marker α-SMA (red) and actin cytoskeleton (phalloidin, green). Nuclei were stained with Hoechst (blue). Direct intercellular contact between organoid tumor cells and CAFs can be observed. Scale bar: 50 μm. **C** Proliferation levels of PDAC organoids measured from culture day 3 to day 8. PDAC organoids in co-culture show significantly higher proliferation levels than in monoculture. Paired t-test, * *P* < 0.05. **D** Schematic overview of the established drug test workflow for PDAC-PDO mono- and PDAC-PDO/CAF co-cultures. **E** Montage of maximum intensity projections as an example of the image data generated from a co-culture model treated with increasing concentration of 5-FU. CAFs (blue) were stained with CellTracker Green CMFDA to be distinguished from PDAC organoids. Hoechst (green) and propidium iodide (red) were used to stain the nuclei of all and dead cells, respectively. **F** Dose-response curves for cell death (left) and proliferation (right) were computed individually for PDOs and CAFs. Area under the curve (AUC, AUCpi) and maximum response values (max. Death, max. PI) for cell death and proliferation inhibition were used to evaluate drug responses

For all five PDO/CAF pairs, we established direct 3D co-culture models (Fig. 1A). For this, matched organoids and CAFs were combined at a 1:1 ratio and were cultured in a 3D matrix composed of Matrigel and collagen I. Direct physical contact of organoids and CAFs was confirmed by fluorescence staining (Fig. 1B). In line with extensive evidence that CAFs promote proliferation of tumor cells in 3D culture models [34], we observed an enhancing effect of CAFs on PDAC organoid proliferation in 4 out of 5 matched co-cultures (Fig. 1C).

### Cancer-associated fibroblasts increase drug resistance of patient-matched pancreatic tumor organoids

To assess the impact of patient-matched CAFs on chemosensitivity of tumor organoids, we used the image-based drug assay DeathPro [22], which allows to evaluate independently drug-induced cell death and proliferation inhibition (PI, Fig. 1D). Sensitivity of PDOs to three chemotherapeutic drugs frequently used in the clinical treatment of PDAC, i.e., gemcitabine, 5-FU and paclitaxel, was evaluated for PDO mono- and PDO/CAF co-cultures. To discriminate CAFs from organoids in the confocal images generated by the DeathPro workflow, CAFs were stained with a cell tracker before setting up the co-cultures (Fig. 1D). By overlaying Hoechst, propidium iodide and the cell tracker signals, individual dose response curves for organoids and CAFs were generated (Fig. 1F).

We observed high reproducibility of the response curves (Supplementary Fig. S1A) and strong correlations (Pearson R > 0.78) for death (AUC, max. death) and proliferation inhibition (AUCpi, max. PI) parameters between independent replicates (Supplementary Fig. S1B). Hoechst and propidium iodide staining had no cytotoxic effects on PDOs or CAFs (Supplementary Fig. S1C and D). In control conditions without drug treatment, the enhancing effect of CAFs on organoid proliferation resulted in an average of 1.3-fold increase in relative growth of organoids in co-culture compared to monoculture (*P* < 0.05; Fig. 1C). Basal levels of cell death in organoids under control conditions did not significantly differ between mono- and co-culture (Supplementary Fig. S1E).

**Fig. 2:**
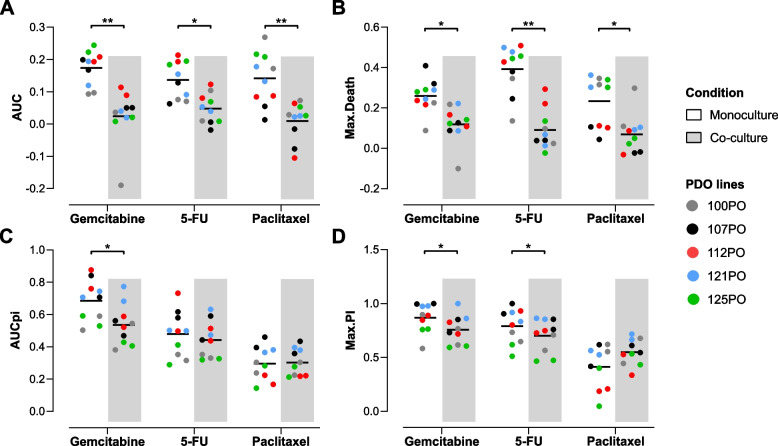
Decreased drug sensitivity of organoids in co-culture with matched CAFs. **A**, **B** Cell death of PDAC organoids induced by gemcitabine, 5-FU and paclitaxel was significantly reduced when co-cultured with CAFs. The mean values of the two independent replicates for each line are displayed. Paired t-test, * *P* < 0.05, ** *P* < 0.01. **C**, **D** Proliferation inhibition induced by gemcitabine in PDAC organoids was significantly reduced in co-culture (AUCpi, max. PI). For 5-FU, a significantly lower max. PI was observed for PDAC organoids in co-culture. The mean values of the two independent replicates for each line are displayed. Paired t-test, * *P* < 0.05

Cell death induced by gemcitabine, 5-FU and paclitaxel treatments was significantly higher in PDO monocultures than in PDO/CAF co-cultures (Fig. 2A and B). Both AUC and max. Death values were on average significantly lower in co-culture conditions, indicating that the presence of CAFs resulted in increased resistance of organoids to the cytotoxic effects of chemotherapy treatments. The extent of this effect was, however, patient and drug specific, suggestive of heterogeneity in the impact of tumor-stroma interactions on chemoresistance mechanisms. PDOs in co-culture also displayed reduced proliferation inhibition (AUCpi and max. PI) induced by gemcitabine (Fig. 2C and D). For 5-FU, only a slight but significant decrease in max. PI was observed. No significant difference was found in organoid proliferation inhibition in the presence of CAFs after paclitaxel treatment (Fig. 2C and D). Worth noting, paclitaxel was less effective at inhibiting organoid proliferation than gemcitabine or 5-FU (Fig. 2C, AUCpi values).

CAFs themselves showed almost no proliferation in 3D co-cultures (Supplementary Fig. S1F, control). Only 5-FU treatment induced a significant increment in CAF death levels (*P* < 0.05; Supplementary Fig. S1G).

In sum, our results show that CAFs exert a protective effect against the cytotoxic effects of gemcitabine, 5-FU and paclitaxel on PDAC-PDOs, indicating a direct contribution to tumor cell drug resistance. These results further highlight the relevance of heterotypic culture models for personalized in vitro drug testing.

### Increased expression of inflammatory pathways in CAFs co-cultured with PDAC organoids

**Fig. 3:**
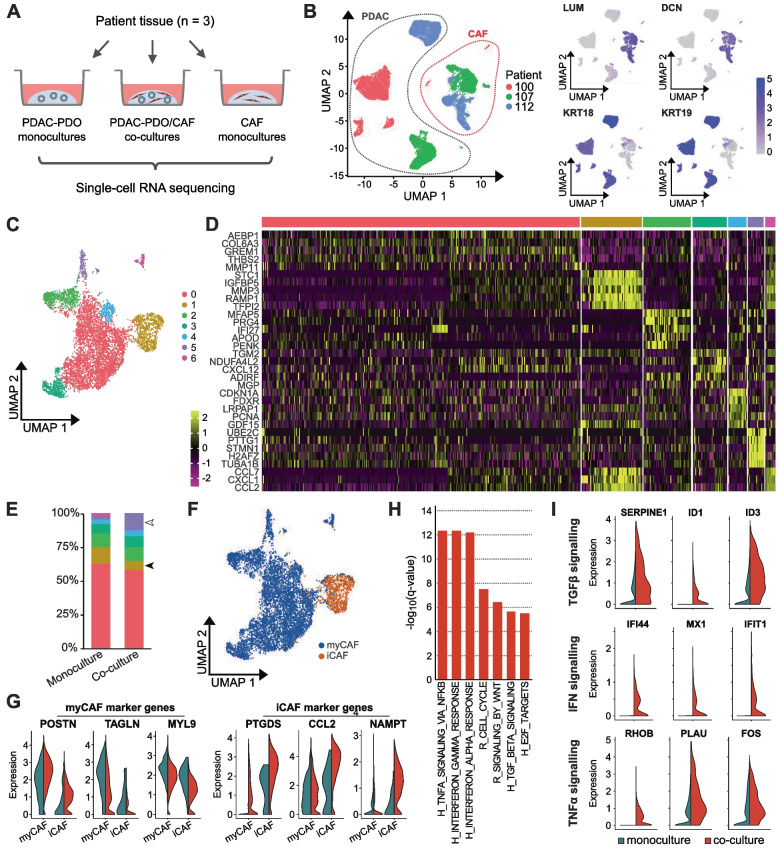
Inflammatory pathways are upregulated in CAFs after co-culture with PDAC organoids. **A** Overview of the samples used for single-cell RNA sequencing. **B** UMAP representation of all single-cell transcriptomes. Dotted lines indicate CAF and PDAC cells, which were distinguished by the expression of known marker genes. LUM and DCN expression identifies CAFs, while KRT18 and KRT19 expression identifies PDAC tumor cells. **C** Integrated UMAP representation of CAFs from monoculture and co-culture samples, with seven clusters distinguished by Louvain clustering. **D** Single-cell expression of the top five enriched genes for each CAF cluster in (C). **E** Distribution of CAFs among the seven clusters, colored as in (C), in monoculture and co-culture samples. The proportion of cells in cluster 1, with an immune response signature, is decreased in co-cultures (filled arrowhead); the proportion of cells in cluster 5, expressing cell cycle related genes, is increased in co-cultures (unfilled arrowhead). **F** Distribution of iCAF-like and myCAF-like cells, as identified by principal component analysis (Methods and Supplementary Fig. S2D), shown on the same UMAP as in (C). **G** Expression of iCAF and myCAF marker genes in the iCAF-like and myCAF-like populations identified in (F), comparing monocultures (blue) to co-cultures (red). **H** Selected Hallmark (H) and Reactome (R) pathways upregulated in CAFs in co-cultures compared to monocultures. **I** Expression of genes involved in TGFβ, IFN and TNFα signaling in CAFs in monocultures (blue) and co-cultures (red) showing increased expression of genes in co-culture conditions

To gain insight into the mechanisms behind the increased chemoresistance of PDAC organoids triggered by CAFs, we performed scRNA-seq to identify transcriptional changes induced in PDO/CAF co-cultures. Three pairs of organoids and patient-matched CAFs (lines 100, 107 and 112) were sequenced after 5 days in 3D mono- and co-culture (Fig. 3A). A total of 13.235 PDAC cells and 7.356 CAFs were sequenced and analyzed (Supplementary Fig. S2A and B). Organoid cells were identified by the expression of the tumor markers KRT18 and KRT19 whereas CAFs were characterized by DCN and LUM expression (Fig. 3B). Patient-specific clustering was observed for organoids and CAF populations, indicating transcriptional heterogeneity between the individual samples (Fig. 3B).

Clustering of all sequenced CAFs distinguished seven transcriptional clusters (Fig. 3C, D and Supplementary Fig. S2C), with distinct functional identities, as indicated by functional enrichment analysis for gene ontology (GO) terms. The largest cluster, cluster 0, was functionally characterized by expression of genes related to extracellular matrix and epithelial to mesenchymal transition. In co-culture, we observed an increase in the proportion of CAFs from cluster 5 (Fisher’s exact test, *p* < 0.0001; Fig. 3E), which was characterized by a higher expression of genes related to cell cycle, suggesting a potential increase of CAF proliferation in co-culture. This increase was consistent across all three patient samples (Supplementary Fig. S2C). On the other hand, contribution of cluster 1, related to inflammation, locomotion and TNF-α signaling, decreased in co-culture (Fisher’s exact test, *p* < 0.0001; Fig. 3E), although this was restricted to case 112 at the individual sample level (Supplementary Fig. S2C). However, we note that these proportions based on scRNA-seq data may not fully reflect cell numbers in cultures, due to possible differences in dissociation efficiency between CAF subtypes.

The two CAF subtypes previously described [20], myCAFs and iCAFs, were identified as CAF subpopulations. Principal component analysis on iCAF and myCAF marker genes [27] for all sequenced CAFs resolved 2 distinct transcriptional clusters (Supplementary Fig. S2D). 88.5% of the CAF population corresponded to myCAFs (6509 cells), whereas 11.5% were classified as iCAFs (847 cells). iCAFs corresponded almost exclusively to the inflammation-related cluster 1 (Fig. 3F). Expression levels of iCAF marker genes were higher in the co-culture setting than in monocultures (Fig. 3G).

We further compared CAFs in mono- and co-culture by gene set enrichment analysis of differentially expressed genes across all cell clusters. Two of the significantly enriched gene sets in co-culture were associated with proliferation (REACTOME_CELL_CYCLE, HALLMARK_E2F_TARGETS; Fig. 3H), which is in line with the increased proportion of the cell cycle related cluster 5. Remarkably, among the top enriched gene sets in co-culture we found hallmark gene sets related to inflammation (TNFα signaling via NFκB, interferon gamma and alpha responses and TGFβ signaling, Fig. 3H and I).

Altogether, these results indicate that co-culture with PDAC organoids induces a pro-inflammatory phenotype in CAFs, which might drive the enhanced chemo-resistance in tumor cells and could be amenable to molecular targeting.

### PDAC organoids display increased expression of EMT genes in 3D co-culture with CAFs

**Fig. 4:**
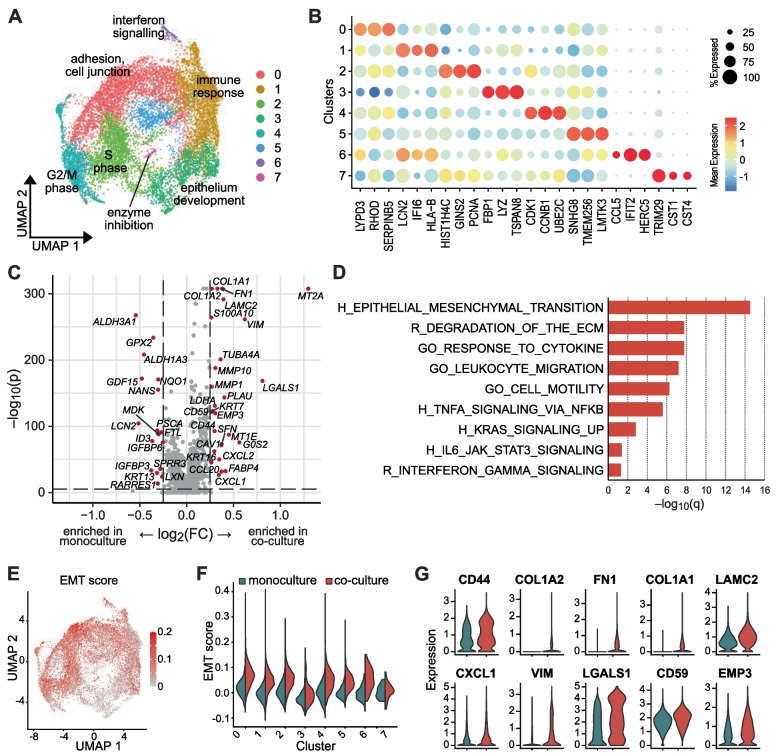
Increased EMT in PDAC organoid cells induced by CAFs. **A** Integrated UMAP representation of PDAC organoid cells from monoculture and co-culture samples, with eight clusters distinguished by Louvain clustering. Keywords indicate functional cluster identities based on GO term analysis. **B** Expression across all PDAC organoid clusters of genes specifically enriched in one cluster. **C** Volcano plot depicting the fold change and significance of genes differentially expressed in PDAC organoid cells in monocultures compared to co-cultures. **D** Selected Hallmark (H), Reactome (R) and GO gene sets upregulated in PDAC organoid cells in co-cultures compared to monocultures. **E** Distribution of EMT scores in PDAC organoid cells, using the same UMAP representation as in (A). **F** Distribution of EMT scores by cluster in PDAC organoid cells in monocultures (blue) and co-cultures (red). **G** Expression of EMT-related genes in PDAC organoid cells in monocultures (blue) and co-cultures (red)

Eight transcriptional clusters were identified for all sequenced PDAC-PDO cells (Fig. 4A, B, Supplementary Fig. S3B and C). The proportion of each cluster differed between organoid lines, reflecting patient-specific transcriptional heterogeneity. For instance, cluster 0, characterized by expression of genes related to adhesion and cell junctions, was the predominant cluster of line 100PO, whereas the immune response related cluster 1 represented most cells in line 107PO (Supplementary Fig. S3A). No predominant cluster was identified in line 112PO (Supplementary Fig. S3A). Interestingly, we did not observe significant changes in the proliferation score for any of the identified transcriptional clusters of PDAC organoid cells in co-culture compared with monoculture (Supplementary Fig. S3D).

We further characterized PDO cells in terms of the two major transcriptional subtypes in PDAC, namely classical and basal-like subtypes, by scoring the expression of each subtype gene signatures at the single-cell level. Organoid line 100PO comprised predominantly cells with higher basal-like scores, whereas 107PO and 112PO showed higher classical scores (Supplementary Fig. S3E). No significant differences in the subtype scores were observed between mono- and co-culture conditions, indicating that the presence of CAFs did not induce a shift in the subtype state of the tumor cells in our model (Supplementary Fig. S3E and F).

To assess the transcriptional changes induced by co-culture with CAFs, we analyzed the differentially expressed genes between PDAC organoids cells in mono- and co-culture conditions (Fig. 4C). Several genes associated with epithelial-to-mesenchymal transition such as COL1A1, COL1A2, FN1, LAMC2 and VIM were upregulated in co-cultured organoid cells. Consistently, Gene Set Enrichment Analysis (GSEA) identified HALLMARK_EPITHELIAL_MESENCHYMAL_TRANSITION as the most significantly enriched gene set in the co-culture settings (Fig. 4D). Additionally, gene sets associated with inflammation such as HALLMARK_TNFA_SIGNALING_VIA_NFKB, HALLMARK_IL6_JAK_STAT3_SIGNALING or REACTOME_ INTERFERON_GAMMA_SIGNALING were also found to be enriched in organoid cells co-cultured with CAFs.

**Fig. 5:**
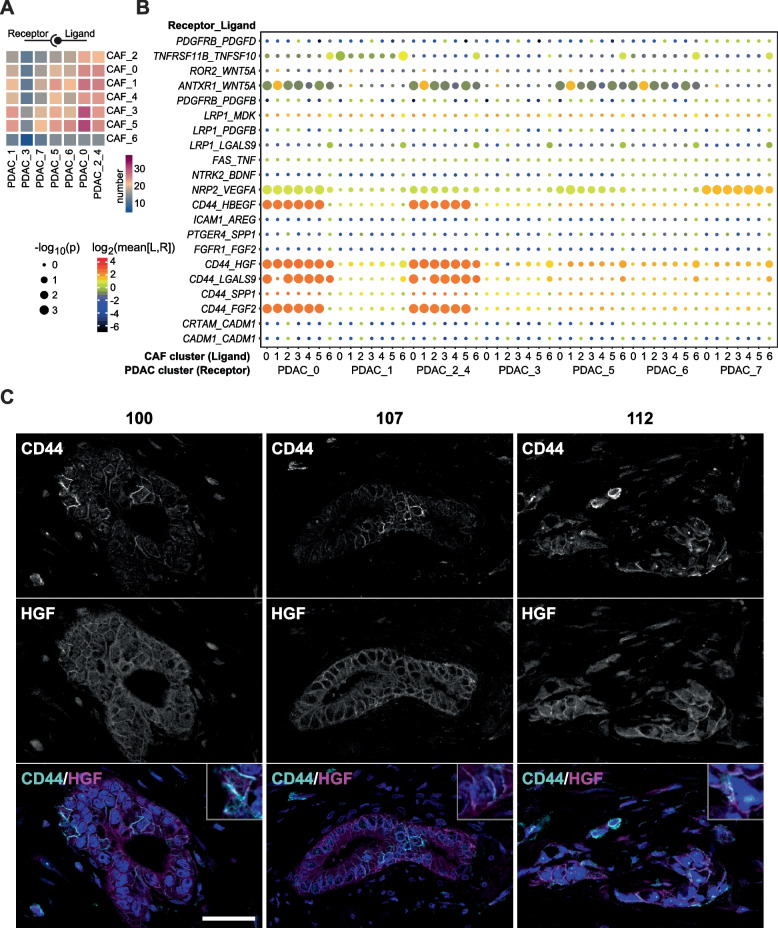
Potential receptor-ligand interactions between PDAC organoids and CAFs. **A** Number of potential ligand-receptor interactions between the different CAF and PDAC organoids cell clusters. **B** Potential receptor-ligand interactions between PDAC organoid and CAF cell clusters with a known or presumed role in EMT. Shown are only interactions where CAF cells present a ligand to the receptor expressed by PDAC organoid cells. **C** Immunofluorescence staining for CD44 (cyan) and HGF (magenta) of the parental tumor tissues of PDO/CAF lines 100, 107 and 112 confirms co-localization of a predicted receptor-ligand interaction in vivo. Single channels and composite are shown. DAPI stained nuclei are depicted in blue. Scale bar: 50 μm

Further focusing on the EMT signature of the identified transcriptional clusters of PDAC cells, we found an increased EMT expression score for almost all clusters in co-culture conditions (Fig. 4E and F), consistent with the result of the GSEA. Expression of several individual EMT-related genes was also upregulated in co-culture (Fig. 4G). Using CellPhoneDB, an open repository of ligand-receptor interactions [32], we were able to detect potential intercellular receptor-ligand interactions between PDO cells (receptor) and CAFs (ligand) (Fig. 5A). In general, most interactions took place between organoid cells in cluster 0 and CAFs. Since cluster 0 was characterized by genes involved in adhesion and cell junctions, this suggests direct intercellular interactions. Additionally, several receptor-ligand interactions related to EMT were identified between PDAC organoid cells and CAFs (Fig. 5B) such as CD44-HGF, CD44-LGALS9 or CD44-FGF2. Most of these EMT-related interactions occurred between CAFs and PDAC organoid cells in cluster 0 as well as clusters 2 and 4 representing cycling PDAC cells (2: S-Phase, 4, G2/M-Phase). Spatial proximity and co-localization of the receptor-ligand interaction pair CD44-HGF could be confirmed in the original tissues by immunofluorescence staining (Fig. 5C), supporting the in vivo relevance of tumor-stroma interactions occurring in our co-culture model.

Taken together, these findings indicate that intercellular interactions between CAFs and epithelial cancer cells are reproduced in our co-culture system, leading to the upregulation of EMT in PDAC organoid cells and increased chemoresistance.

## Discussion

Initial studies have demonstrated that PDAC organoids reflect the genetic diversity of the original tumors and that drug sensitivity of organoid lines correlates with clinical response, providing a first indication of their potential for personalized medicine [16, 19]. However, the absence of stromal components in these models has not yet allowed to evaluate the influence of stromal activity and signaling on chemosensitivity and precludes screening of drugs targeting stromal elements or tumor-stroma interactions.

In this study, we established direct co-cultures of patient-matched PDAC organoids and CAFs and showed that CAFs promoted epithelial cancer cell proliferation as well as resistance of cancer cells to gemcitabine, 5-FU and paclitaxel. The use of the image-based DeathPro assay [22] allowed us to overcome the challenge of differentiating cell type-specific drug responses. Common in vitro viability assays such as MTT or CellTiter Glo are not capable of differentiating drug responses of stromal and tumor cells when directly co-cultured. Hence, in addition to discerning specific tumor cell responses, this system allows monitoring the specific effect of drug treatment on CAFs, providing an excellent tool to study combination therapies targeting tumor and stromal cells.

Previous studies have reported enhanced chemoresistance of primary human PDAC cells in the presence of unmatched stromal cells in 3D tumor spheroid and bioengineered models [35, 36]. Similarly, Tsai et al. reported an increase in IC50 after gemcitabine treatment of one patient-derived organoid line in co-culture with CAFs [21]. Our work extends on these observations in a patient-specific manner, highlighting the feasibility and relevance of complex organoid models for ex vivo drug response prediction in the clinical setting. In our scRNA-seq data, we observed unsupervised clustering of organoid tumor cells as well as of CAFs by patient of origin; patient-specific heterogeneity is thus preserved in both cell populations. These results accentuate the need and potential to incorporate patient-matched stromal cells into organoids to faithfully model tumor diversity across patients. In our experience, the limiting factor for clinical application of our co-culture model would be the time necessary to reach sufficient purity and biomass of the organoid cultures. Successful establishment and expansion of organoid lines was accomplished in 50% of the cases and downstream applications could be performed after a median of 40 days. Considering that adjuvant therapy is commonly started between 6 and 12 weeks after resection, drug response prediction based on our model could inform first-line adjuvant treatment for a subset of patients while results could be available to inform second-line treatments for most of these patients. Additionally, successful generation of PDAC-PDOs [18, 38, 39] as well as a PDO/CAF pair [37] from minimal samples such as fine-needle biopsies have been previously reported, supporting a potential clinical application in the neoadjuvant or palliative settings. Further optimization and patient-specific tailoring of organoid culture conditions to accelerate organoid expansion could be therefore highly beneficial. Recently, Harryvan et al. [37] described a pancreatic mini-tumor model established from patient-derived organoids and CAFs, which was able to model the characteristic desmoplastic reaction observed in PDAC tumors. In their study, CAF activation and proliferation was induced by activation of transforming growth factor and platelet-derived growth factor β signaling pathways via modulation of culture media components, supporting the potential of fine-tuning PDO/CAF co-culture conditions to model even more closely tumor drug response.

Our scRNA-seq analyses also revealed transcriptional changes towards an EMT phenotype in co-cultured organoid cells, consistent with the results reported by Ligorio and colleagues for 2D co-cultures of patient-derived PDAC cell lines derived from metastatic ascites and unmatched CAFs [40]. This observation is also in line with previous reports of EMT induction in PDAC cell lines after indirect co-culture with pancreatic stellate cells (PSCs) cells [41] or exposure to PSC-condition medium [42]. In vivo, orthotopically grafted human organoids surrounded by a stromal microenvironment evidenced upregulation of EMT signaling relative to organoids engrafted intraductally, emphasizing that induction of EMT in tumor cells by the stromal microenvironment is a relevant process in vivo [43]. While the role of EMT in driving PDAC chemoresistance is widely recognized [44, 45], our results indicate CAF-mediated induction of EMT in organoids as one of the mechanisms contributing to their chemo-protectant effect in our co-culture system.

Despite changes in EMT, we did not observe shifts in the transcriptional subtype signatures of the tumor cells under our culture conditions. Although proliferation of organoids was enhanced in co-cultures, proliferation scores derived from the scRNA-seq analyses were not significantly different between mono- and co-cultures. Similarly, the increment in the proportion of the CAF cluster expressing cell cycle related genes observed in co-cultures was not accompanied by enhanced CAF proliferation as determined by DeathPro. It is worth noting that in our scRNA-seq data we identified considerably lower numbers of CAFs in co-culture compared to CAF monoculture samples. It is thus conceivable that the observed changes in CAF cluster proportions might be due to differential loss of CAF cells in the co-culture setting during sample processing due to, for example, preferential survival of cells with rounded shape or exclusion of CAF-tumor cell doublets. A challenging recovery of CAFs has also been reported for primary pancreatic tumor samples [27], raising awareness that single cell analyses of CAFs might be generally affected by technical biases during dissociation.

Depletion of tumor stroma results in more aggressive tumors and reduced survival in preclinical models [46, 47] and stroma-depleting agents have shown no benefit in clinical trials [48]. Targeting of specific tumor-stromal cells interactions, in contrast, has become a promising therapeutic approach for PDAC [49]. Results from our scRNA-seq analyses identified several potential interactions involving the cancer stem cell marker CD44 with ligands secreted by CAFs such as HGF, HBEGF, FGF2 and LGALS9. We could also show co-localization of CD44 and HGF in the parental tumor tissues, validating the relevance of predicted interactions from our co-culture model in the in vivo context. CD44 signaling has also been shown to promote stemness via a SPP1-CD44 axis [50] and high expression of CD44 has been associated with poor prognosis in PDAC [51]. Evidence from patient-derived xenografts shows that CD44^+^ cells are the source of PDAC relapse after gemcitabine treatment, rendering CD44 a promising therapeutic target against recurrent disease [52].

## Conclusions

Altogether, our study demonstrates increased chemoresistance of PDAC organoids in co-culture with patient-matched CAFs, emphasizing the relevance of complex co-culture models for personalized medicine applications. This also opens the possibility to investigate efficacy and mode of action for drugs targeting the tumor microenvironment in a patient-specific manner. Hence, our work provides evidence of the feasibility of modeling patient-specific tumor-stroma interactions for target discovery and drug testing in a high-throughput amenable 3D culture setting.

## Supplementary Information

**Additional file 1 **: **Supplementary Figures.** Supplementary Figs. S1 to S3.

**Additional file 2 **: **Supplementary Table S1.** Additional information of scRNA-seq data: maximum number of counts per gene and a maximum fraction of mitochondrial reads.

## Data Availability

Single-cell sequencing data will be deposited at the European Genome-Phenome Archive (EGA) under accession number EGAS00001006661. All materials will be available upon request through a material transfer agreement.

## Abbreviations

CAFs: Cancer-associated fibroblasts
PDAC: Pancreatic ductal adenocarcinoma
3D: Three dimensional
EMT: Epithelial-to-mesenchymal transition
ECM: Extracellular matrix
PDOs: Patient-derived PDAC organoids
5-FU: 5-fluorouracil
scRNA-seq: Single cell RNA-sequencing
iCAFs: Inflammatory CAFs
myCAFs: Myofibroblastic CAFs
AUC: Area under the curve
PI: Proliferation inhibition
GSEA: Gene Set Enrichment Analysis
PSCs: Pancreatic stellate cells
