# Supplementary material for: Patient-specific modeling of stroma-mediated chemoresistance of pancreatic cancer using a three-dimensional organoid-fibroblast co-culture system
Source: J Exp Clin Cancer Res. 2022 Oct 22;41:312. doi: 10.1186/s13046-022-02519-7 (PMC9588250; doi:10.1186/s13046-022-02519-7)
Supplement: Supplementary file 1 — Additional file 1 : Supplementary Figures. Supplementary Figs. S1 to S3. [file 13046_2022_2519_MOESM1_ESM.pdf]

Supplementary Figures

Supplementary Figure S1

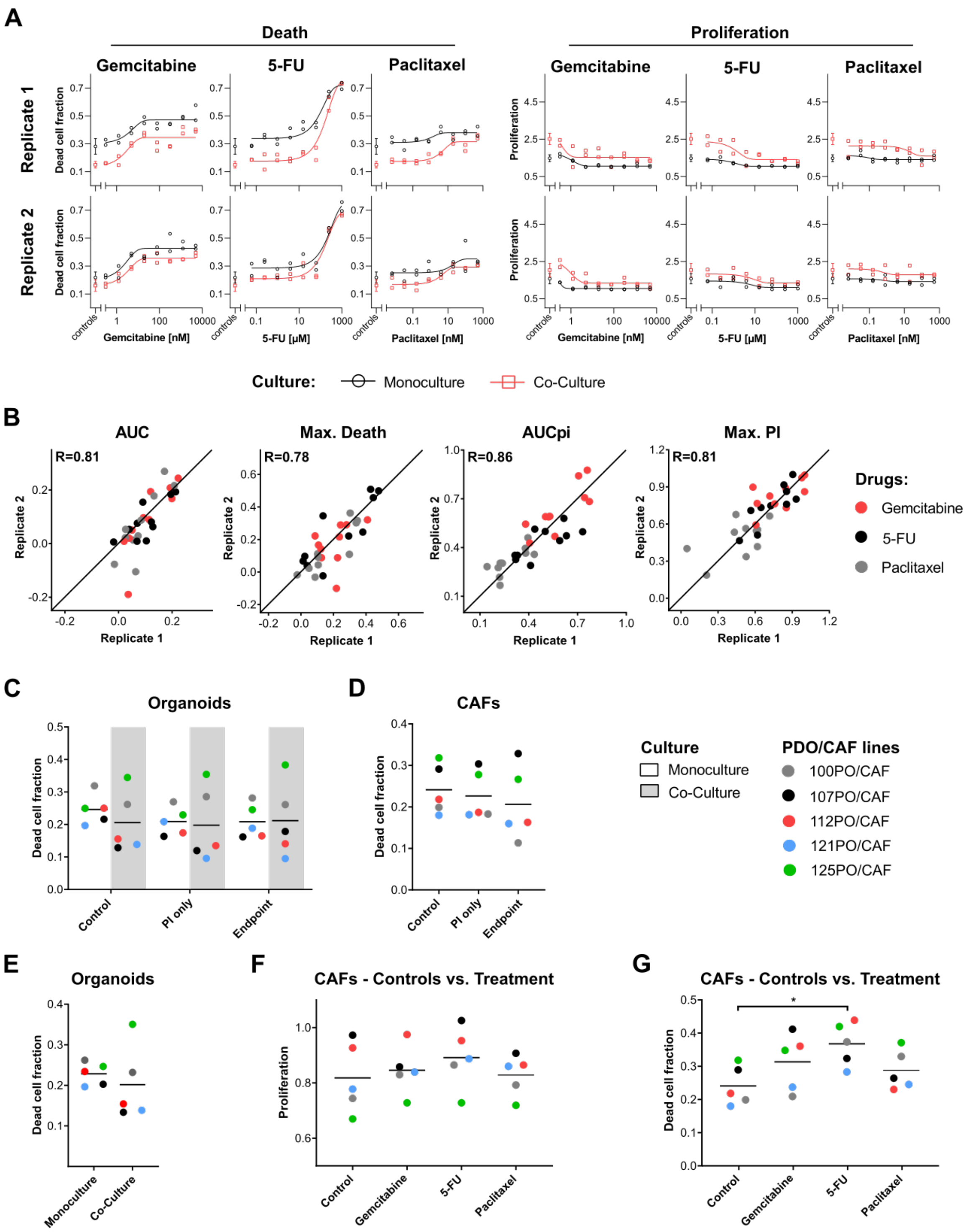

Supplementary Figure S1. A) Example of the dose response curves generated after drug testing for 112PO in mono- and co-culture settings. **B)** Pearson correlation of the assessed assay parameters

between independent replicates. **C)** Hoechst and propidium iodide (PI) staining had no cytotoxic effect on PDAC organoids. Control samples (stained with Hoechst + PI on day 3 and day 8) were compared with only PI-stained (day 3: PI, day 8: Hoechst + PI) or endpoint-stained samples (day 3: no staining, day 8: Hoechst + PI). **D)** Hoechst and propidium iodide (PI) staining had no cytotoxic effect on CAFs. Control samples (stained with Hoechst + PI on day 3 and day 8) were compared with only PI-stained samples (day 3: PI, day 8: Hoechst + PI) or endpoint-stained (day 3: no staining, day 8: Hoechst + PI). **E)** Cell death of untreated PDAC organoids (controls) after 8 days in culture. **F-G)** Effect of gemcitabine, 5-FU and paclitaxel on proliferation (**F**) and cell death (**G**) of CAFs in co-culture with PDOs. Dunnett's test, * $P < 0.05$.

Supplementary Figure S2

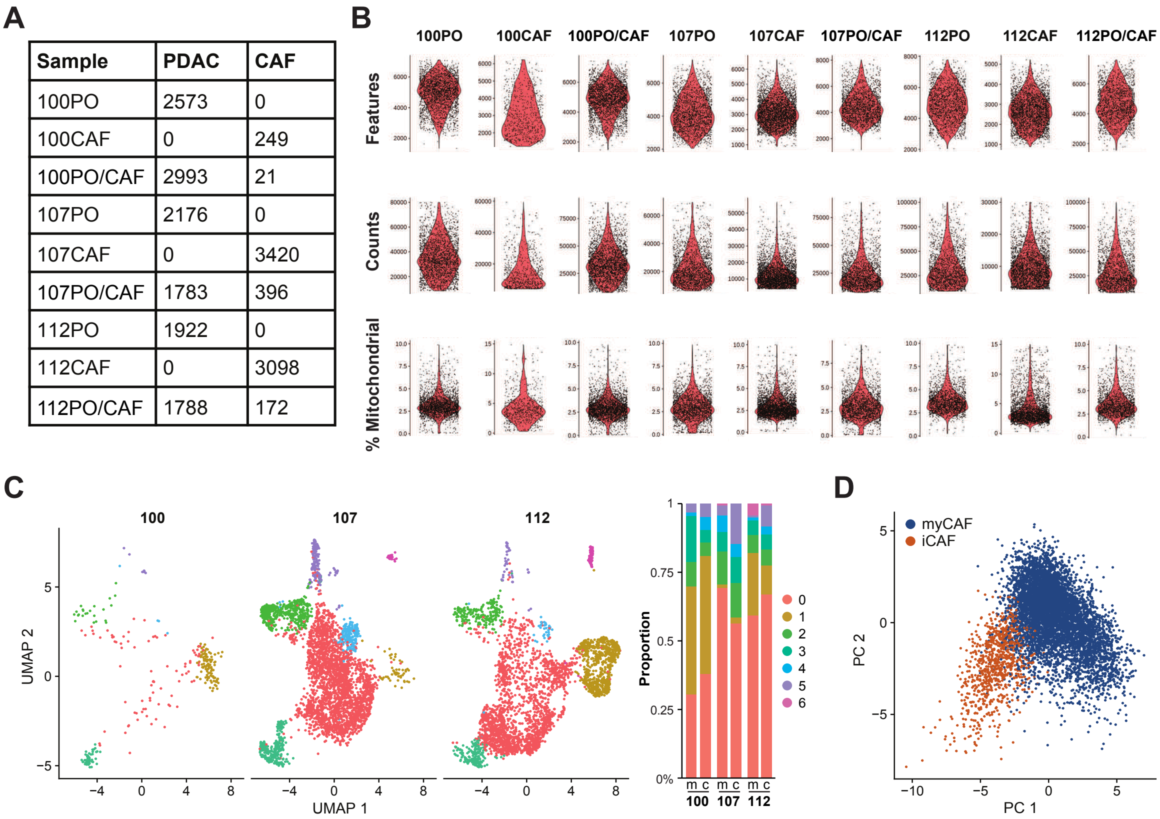

Sample	PDAC	CAF
100PO	2573	0
100CAF	0	249
100PO/CAF	2993	21
107PO	2176	0
107CAF	0	3420
107PO/CAF	1783	396
112PO	1922	0
112CAF	0	3098
112PO/CAF	1788	172

Supplementary Figure S2. A) Number of PDO and CAF cells identified in each sample. **B)** Number of features (genes), number of RNA counts, and percentage of mitochondrial counts identified in each sample. **C)** Left: Distribution of CAF cells among clusters, shown as UMAP split by patient origin. Right: Bar plot showing CAF cluster proportions split by patient origin and sample type (m: monoculture, c: co-culture). **D)** PCA projection of CAF cells from mono- and co-culture samples, with PCA computed on iCAF and myCAF marker genes.

Supplementary Figure S3

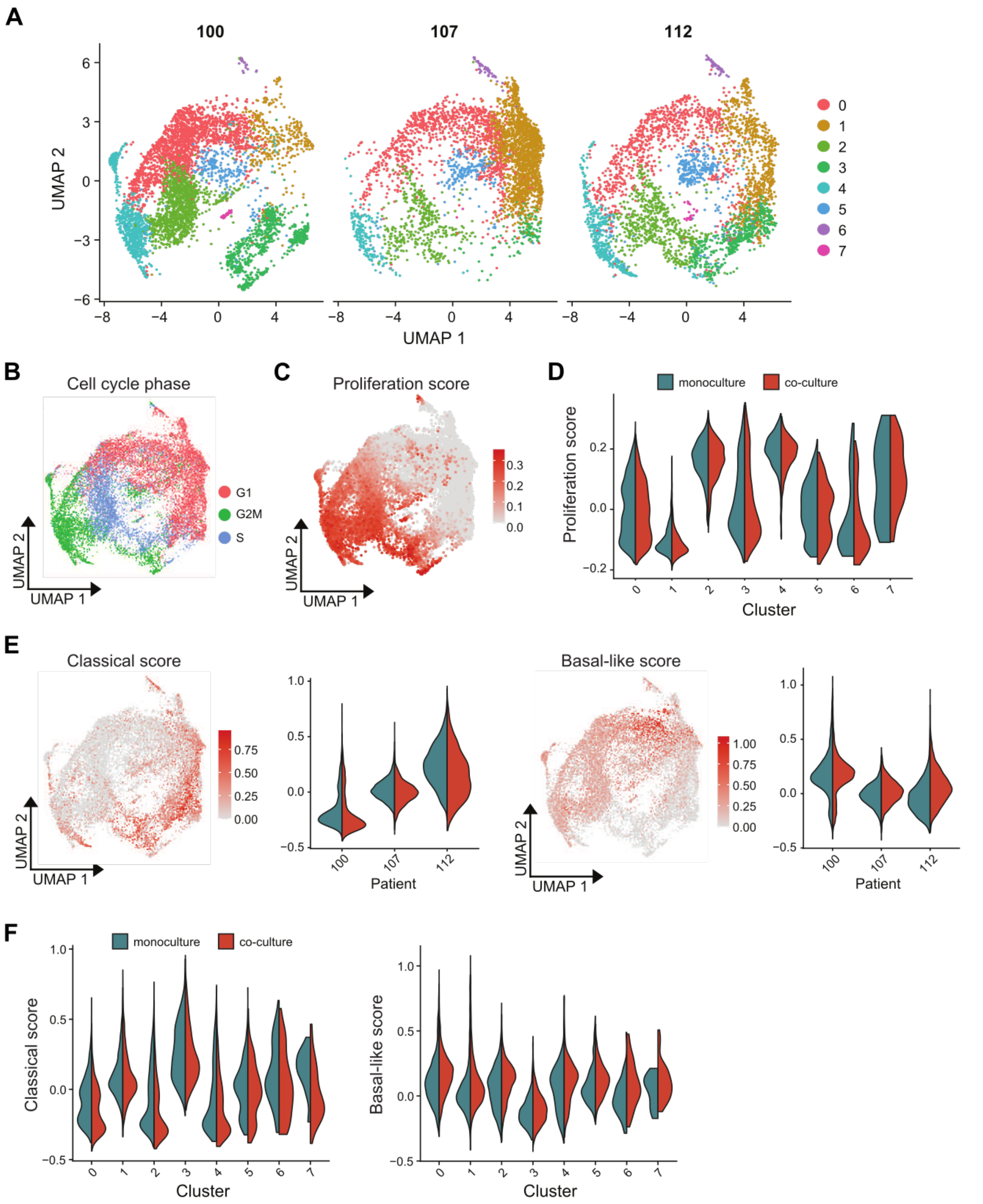

Supplementary Figure S3. A) Distribution of PDAC-PDO cells among clusters, split by patient origin. **B)** Assignment of PDAC-PDO cells to G1, G2/M or S phase based on marker gene expression, using the same UMAP representation as in Fig. 3A. **C)** Distribution of proliferation scores (gene set:

HALLMARK_E2F_TARGETS) across PDAC-PDO cells. **D)** Distribution of proliferation scores by cluster in PDAC-PDO cells in monocultures (blue) and co-cultures (red). **E)** UMAPs: Distribution of classical and basal-like subtype scores across PDAC-PDO cells. Violin plots: Distribution of classical and basal-like subtype scores in monocultures (blue) and co-cultures (red), split by patient origin. **F)** Distribution of classical and basal-like subtype scores across organoid cell clusters in monocultures (blue) and co-cultures (red).
