# Supplementary material for: Patient-specific modeling of stroma-mediated chemoresistance of pancreatic cancer using a three-dimensional organoid-fibroblast co-culture system
Source: J Exp Clin Cancer Res. 2022 Oct 22;41:312. doi: 10.1186/s13046-022-02519-7 (PMC9588250; doi:10.1186/s13046-022-02519-7)
Supplement: Supplementary file 2 — Additional file 2 : Supplementary Table S1. Additional information of scRNA-seq data: maximum number of counts per gene and a maximum fraction of mitochondrial reads. [file 13046_2022_2519_MOESM2_ESM.pdf]

Supplementary Table S1

Additional information of scRNA-seq data: maximum number of counts per gene and maximum fraction of mitochondrial reads.

Sample ID	Maximum RNA counts per gene	Maximum percentage of mitochondrial reads
**100PO**	80000	10
**100CAF**	60000	15
**100PO/CAF**	90000	10
**107PO**	70000	10
**107CAF**	60000	10
**107PO/CAF**	90000	10
**112PO**	100000	10
**112CAF**	30000	15
**112PO/CAF**	100000	10
